# Changes of Basilar Artery in Patients With Migraine: A Case‐Control Study Based on 3T MRI

**DOI:** 10.1002/brb3.70955

**Published:** 2025-10-20

**Authors:** Mantian Zhang, Xing Li, Yanliang Mei, Xiaoshuang Li, Zhi Guo, Chenchen Ma, Yumei Gu, Feng Guo, Zhonghua Xiong, Peng Zhang, Dong Qiu, Tianshuang Gao, Geyu Liu, Yaqing Zhang, Xueying Yu, Yuesong Pan, Binbin Sui, Yonggang Wang, Hefei Tang

**Affiliations:** ^1^ Headache Center, Department of Neurology, Beijing Tiantan Hospital Capital Medical University Beijing China; ^2^ Department of Neurology The First Hospital of Fangshan District Beijing China; ^3^ Department of Neurology Third Affiliated Hospital of Soochow University Changzhou China; ^4^ Department of Neurology The First Affiliated Hospital of Guangdong Pharmaceutical University Guangzhou Guangdong China; ^5^ Department of Neurology The First People's Hospital of Guangyuan City Guangyuan Sichuan China; ^6^ Department of Neurology, Beijing Tiantan Hospital Capital Medical University Beijing China; ^7^ China National Clinical Research Center for Neurological Diseases Beijing China; ^8^ Tiantan Neuroimaging Center of Excellence China National Clinical Research Center for Neurological Diseases Beijing China

**Keywords:** biomarker, chronic migraine, magnetic resonance angiography, migraine without aura

## Abstract

**Objective:**

As a neurovascular disorder, migraine currently lacks well‐established macroscopic biomarkers detectable by magnetic resonance angiography (MRA). While the basilar artery (BA) has been implicated in migraine pathophysiology, this relationship remains poorly characterized. This study investigates whether BA morphological parameters could serve as diagnostic biomarkers for migraine and predictive markers for disease progression.

**Methods:**

This study included 41 healthy controls (HCs), 41 episodic migraine (EM) patients, and 95 chronic migraine (CM) patients who completed both MRI examinations and standardized questionnaires. Using established diagnostic criteria for vertebrobasilar dolichoectasia (VBD), we quantified the diameter, length, and height of the BA bifurcation. Furthermore, we measured the superior cerebellar artery (SUCA) outlet angle and basilar artery lateral displacement (BALD). These BA‐derived metrics were subsequently incorporated into multivariable logistic regression models to assess their predictive value for migraine chronification.

**Results:**

No significant differences were found in BA diameter, BA length, BADE, or VBD when comparing either EM or CM groups with HCs. However, both BALD and SUCA outlet angles showed significant intergroup differences. There was a statistically significant difference in BADE between EM and CM. In the logistic regression, migraine was significantly associated with both BALD and SUCA outlet angles. In the multinomial logistic regression analysis, EM was significantly associated with SUCA outlet angle, while CM was significantly associated with both BALD and SUCA outlet angle.

**Conclusions:**

Our data suggest that the reduced SUCA outlet angle may represent a risk factor for migraine and could potentially serve as an imaging biomarker. Additionally, BALD may constitute an independent risk factor for CM and could function as an MRA biomarker for migraine chronicity. Potential indicators related to the basilar artery may influence migraine attacks by affecting hemodynamic changes in migraine pathophysiology.

## Graphical abstract

This single‐center study from China revealed a reduced superior cerebellar artery outlet angle in patients with migraine without aura, as well as lateral displacement of the basilar artery in those with chronic migraine. These findings may provide further evidence supporting the characterization of migraine as a neurovascular disorder.

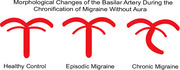

## 1 Introduction

Migraine is a common neurovascular disorder closely related to genetics (GBD 2019 Diseases and Injuries Collaborators 2020; Steiner et al. 2020). Vascular dysfunction plays a pivotal role in its pathophysiology (Ferrari et al. 2022). Recent genome‐wide association studies have identified over 180 migraine‐associated genetic variants enriched in vascular and neuronal tissues. Among these, low‐density lipoprotein receptor‐related protein‐1 (LRP1), a key regulator of vascular integrity (Strickland et al. 2014), is the gene locus most likely associated with migraine, further underscoring the close connection between vasculature and migraine (Bjornsdottir et al. 2023; Gormley et al. 2016; Grangeon et al. 2023).

The involvement of intracranial and extracranial arteries in migraine remains debated. Experimental stimulation and mechanical dilation could induce focal headache, suggesting that intracranial large arteries may contribute to migraine pathogenesis (Nichols et al. 1990; Ray and Wolff 1940). A cross‐sectional study found that migraine attacks are associated with mild intracranial artery dilation but not extracranial dilation. Notably, sumatriptan administration during attacks induces minor but significant circumferential contraction of the basilar artery (BA), while no such effect is observed in the middle cerebral artery or intracranial internal carotid artery (Amin et al. 2013). Meanwhile, a transcranial Doppler study reveals endothelial dysfunction specifically in the BA of migraineurs, even in the absence of systemic endothelial dysfunction, though no differences exist between migraine without aura (MO) and migraine with aura (MA), which may suggest potential abnormalities in the BA (Rajan et al. 2015).

Previous researchers found that individuals with migraine may show reduced outlet angle of the superior cerebellar artery (SUCA) and severe basilar artery lateral displacement (BALD), and notably, this phenomenon was predominantly observed in patients with MA (Hensel et al. 2023; Yang et al. 2022; C. Zhang et al. 2018). Despite differences in the number of participants and the content of the assessments, the overall trend of the results also suggests migraine may be associated with the BA.

However, to our knowledge, the pathophysiology of classic trigeminal neuralgia is attributed to compression of the trigeminal nerve's sensory component by an adjacent artery, most commonly the SUCA (Araya et al. 2020; Cruccu et al. 2020). Recently, Islam et al. (2024) summarized potential common and distinctive hypothalamic mechanisms underlying trigeminal neuropathic pain, migraine, and cluster headache, suggesting that current understanding of neuropathic pain interrelationships may be incomplete. Notably, while BA alterations may represent a shared characteristic of craniofacial pain disorders, previous studies have not established a mechanistic explanation for this association (Hensel et al. 2023; Yang et al. 2022; C. Zhang et al. 2018). Meanwhile, in cerebrovascular diseases, BALD constitutes a component of vertebrobasilar dolichoectasia (VBD), which is characterized by dilation, elongation, and tortuosity of the vertebrobasilar artery (Samim et al. 2016). Although dolichoectasia may affect both anterior and posterior circulations, the posterior circulation, particularly the BA, is most frequently involved (Pico et al. 2007). This suggests that evaluating BA tortuosity in migraine patients may be clinically relevant. The Smoker criteria are widely used for VBD diagnosis, where the laterality score reflects tortuosity, the bifurcation height indicates elongation, and the diameter measures dilation (Gutierrez et al. 2011). Alternatively, another way to evaluate BA is basilar artery dolichoectasia (BADE), which was defined as BA diameter > 4.5 mm, laterality score > 2, or height of bifurcation score > 2 (Zhai et al. 2018).

In summary, while the relationship between migraine and BA abnormalities remains inconclusive, the potential association with BA elongation or tortuosity has not been systematically investigated. The role of BA alterations in migraine pathogenesis remains undetermined. This study aims to further evaluate BA‐related indicators as potential MRA biomarkers for migraine and investigate their associations with migraine onset, progression, and chronicity.

## 2 Methods

### 2.1 Protocol Approvals and Patient Consent

This single‐center, case‐control study was a part of an ongoing clinical trial, the China Headache Disorders Registry Study (CHAIRS, NCT05334927). Before inclusion in the study, all participants signed informed consent under the principles of the Declaration of Helsinki.

### 2.2 Study Population

All patients with migraine who visited the headache department of Beijing Tiantan Hospital with completed brain magnetic resonance imaging (MRI) scanning and a headache questionnaire between January 2021 and September 2024 were recruited. All diagnoses of migraine, based on the International Classification of Headache Disorders 3rd edition (ICHD‐3) (“Headache Classification Committee of the International Headache Society (IHS) The International Classification of Headache Disorders, 3rd Edition,” 2018), were made by at least two professional headache specialists. The inclusion criteria for patients included: (1) age between 18 and 60 years; (2) age of onset less than 50 years; and (3) no prior use of preventive medications. The exclusion criteria for patients included: (1) patients with other types of primary headaches, other neurological diseases, psychiatric disorders, or a history of neurosurgery; (2) poor quality or missing MRI data; (3) pregnant or lactating women; and (4) missing clinical information or questionnaire data. Inclusion criteria for healthy controls (HCs) included: (1) matching the age and gender of the patients and (2) no personal or family history of headache. Exclusion criteria for HCs included: (1) pregnant or lactating women; (2) poor quality or missing MRI data; (3) having neurological or other major systemic diseases; (4) missing clinical information or questionnaire data. The questionnaire that all participants completed included a detailed personal history (encompassing smoking history, alcohol intake history, family history, etc.), a detailed history of headache (including age of onset, location, nature, severity, frequency, triggering factors, past medication usage, etc.), Visual Analogue Scale (VAS), Migraine Disability Assessment Scale (MIDAS), Headache Impact Test‐6 (HIT‐6), Generalized Anxiety Disorder‐7 (GAD‐7), Patient Health Questionnaire‐9 (PHQ‐9), and Pittsburgh Sleep Quality Index (PSQI). Based on previous studies, we considered patients with a PSQI score of ≥ 8 to have sleep disorders (Reilly‐Spong et al. 2013; Yuan et al. 2023).

### 2.3 MRI Acquisition and Assessment of BA

All MRI data were acquired using a 3.0T PET/MR system (SIGNA PET/MR, GE Healthcare), which included sequences such as axial three‐dimensional T1‐weighted brain volume (T1w‐BRAVO), magnetic resonance angiography (MRA), and time‐of‐flight MRA (TOF‐MRA). Participants were instructed to minimize head and neck movements during the MRI scan, stay awake, relax, and keep their eyes closed. Earplugs and foam pads were used to reduce noise from the scanner and any head movements. The detailed acquisition parameters of the sequences are listed in Table S1. The evaluation of the data was conducted simultaneously by two experienced and specially trained neuroradiologists who were blinded to any clinical information about the subjects. Based on the diagnostic criteria of MRI for VBD and BADE, we measured the diameter, length, and height of the BA bifurcation and the lateral position of the BA on T1‐weighted image, MRA, and TOF‐MRA (Samim et al. 2016; Zhai et al. 2018). Based on a previous study, for abnormally shaped BA, BALD is the distance from the center of the maximally curved segment of the vessel to the standard line of the BA (D.‐P. Zhang et al. 2014). To compare with the results of previous articles, we also evaluated the SUCA outlet angle (Hensel et al. 2023).

### 2.4 Statistical Analysis

All statistical data were analyzed using SPSS software for Windows (Version 25.0). The normally and non‐normally distributed data were described using mean ± standard deviation and median with interquartile range, respectively. Categorical variable data were described using numbers with percentages. The independent samples *t*‐test was applied to compare normally distributed data between the groups. The Mann–Whitney *U* test and the chi‐square or Fisher's exact tests were used to compare non‐normally distributed continuous data and categorical variable data, respectively. Subsequently, clinical variables with *p *< 0.05 or those related to migraine, such as age and gender, were included in the binary multivariate logistic regression analysis. We assessed the model fit of the regression model using the Hosmer–Lemeshow test. Finally, we constructed a multinomial logistic regression model to assess the correlation between BA‐related indicators and EM and CM.

## 3 Results

### 3.1 Demographics and Clinical Characteristics

**FIGURE 1:**
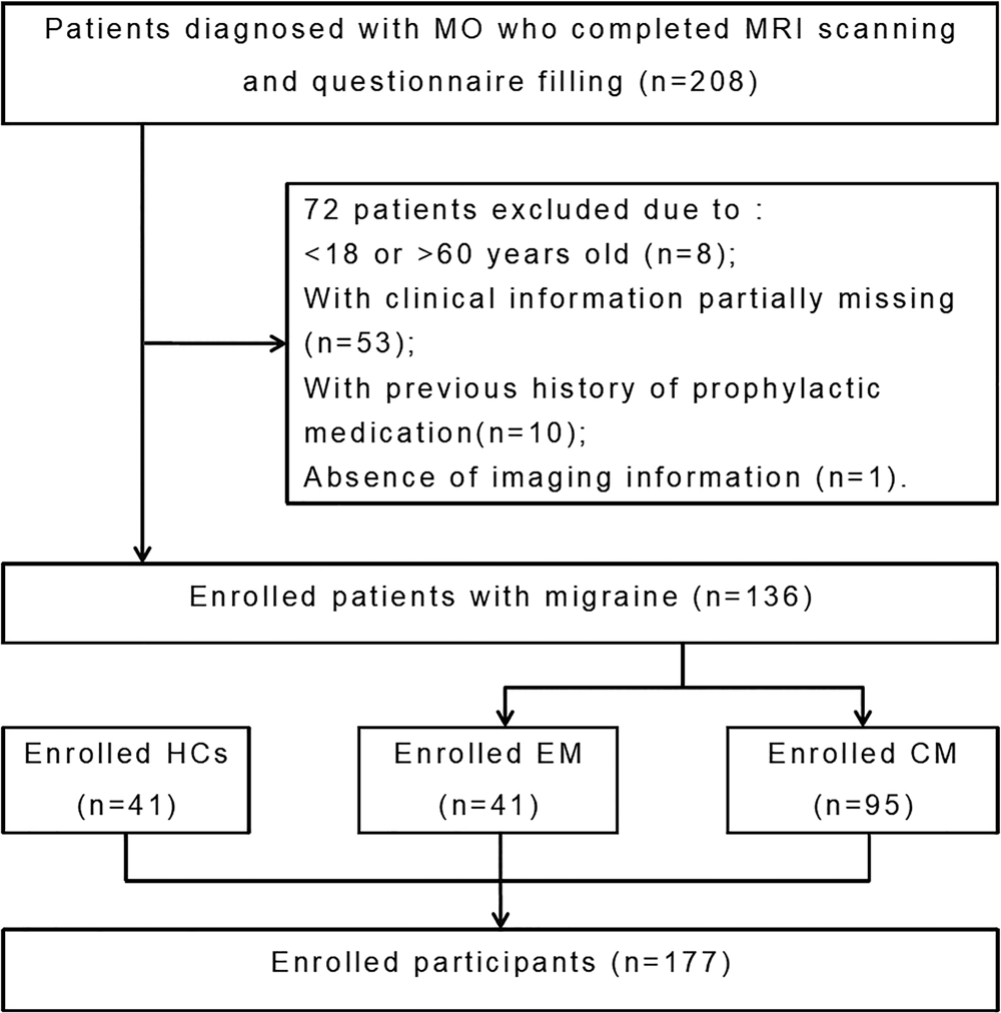
Flowchart of participants’ enrollment. CM, chronic migraine; EM, episodic migraine; HCs, healthy controls; MO, migraine without aura.

As shown in Figure 1, we included 41 HCs and 136 patients with MO, comprising 41 patients with episodic migraine (EM) and 95 with chronic migraine (CM). Detailed demographic information and clinical characteristics of the participants are all presented in Table 1.

**TABLE 1:** Demographics and clinical characteristics of participants.

	**HCs (*n* = 41)**	**EM (*n* = 41)**	**CM (*n* = 95)**	***p* value (HC vs. EM)**	***p* value (HC vs. CM)**	***p* value (EM vs. CM)**
Age, years	38.00 (28.00, 45.00)	39.00 (31.00, 45.50)	42.00 (34.00, 49.00)	0.864^a^	0.058^a^	0.100^a^
Female, *n* (%)	28 (68.29)	31 (75.61)	70 (73.68)	0.461^b^	0.520^b^	0.814^b^
Body mass index, kg/m^2^	23.15 (20.31, 25.02)	22.77 (20.32, 26.26)	22.49 (20.31, 25.83)	0.770^a^	0.864^a^	0.727^a^
Current smoker, *n* (%)	4 (9.76)	7 (17.07)	10 (10.53)	0.517^c^	> 0.999^c^	0.289^b^
Current drinker, *n* (%)	9 (21.95)	8 (19.51)	17 (17.89)	0.785^b^	0.581^b^	0.823^b^
Sleep disturbance, *n* (%)	5 (12.20)	14 (34.15)	65 (68.42)	0.018^b^	< 0.001^b^	< 0.001^b^
BA diameter, mm	3.45 (3.14, 3.74)	3.01 (3.13, 3.92)	3.46 (3.01, 4.25)	0.250^a^	0.423^a^	0.144^a^
BA length, mm	23.30 (21.40, 26.00)	23.37 ± 3.67	24.20 ± 3.05	0.967^a^	0.205^a^	0.177^e^
BALD, mm	0 (0.00, 3.09)	1.92 (0.00, 4.04)	2.58 (1.32, 4.33)	0.107^a^	0.001^a^	0.111^a^
BADE, *n* (%)	2 (4.88)	1 (2.44)	17 (17.89)	> 0.999^c^	0.082^c^	0.030^c^
VBD, *n* (%)	1 (2.44)	0	3 (3.16)	> 0.999^d^	> 0.999^c^	0.554^d^
SUCA outlet angle	159.70 (147.75, 173.50)	141.00 (126.50, 163.00)	143.00 (129.00, 157.00)	0.014^a^	0.001^a^	0.930^a^
Age of onset, years	NA	22.87 ± 9.76	21.83 ± 9.71	NA	NA	0.570^e^
Disease duration, years	NA	14.00 (8.00, 20.00)	20.00 (10.00, 29.00)	NA	NA	0.057^a^
Migraine frequency, d/mo	NA	5.00 (4.00, 10.00)	25.00 (20.00, 30.00)	NA	NA	< 0.001^a^
Medication overuse, *n* (%)	NA	NA	63 (66.32)	NA	NA	NA
MIDAS score (0–270)	NA	35.00 (15.00, 62.50)	105.00 (60.00, 155.00)	NA	NA	< 0.001^a^
VAS score (0–10)	NA	7.00 (6.00, 8.00)	7.00 (6.00, 9.00)	NA	NA	0.184^a^
HIT‐6 score (36–78)	NA	63.00 (56.00, 69.50)	66.00 (62.00, 72.00)	NA	NA	0.012^a^
PHQ‐9 score (0–27)	NA	6.00 (1.00, 9.50)	10.00 (6.00, 16.00)	NA	NA	< 0.001^a^
GAD‐7 score (0–21)	NA	4.00 (0.50, 8.50)	7.00 (3.00, 12.00)	NA	NA	0.010^a^

*Note*: Sleep disturbance means PSQI ≥ 8.

Abbreviations: BA, basilar artery; BADE, basilar artery dolichoectasia; BALD, basilar artery lateral displacement; CM, chronic migraine; d/mo, day per month; EM, episodic migraine; GAD‐7, Generalized Anxiety Disorder‐7; HCs, healthy controls; HIT‐6, Headache Impact Test‐6; MIDAS, Migraine Disability Assessment Scale; NA, not applicable; PHQ‐9, Patient Health Questionnaire‐9; PSQI, Pittsburgh Sleep Quality Index; SUCA, superior cerebellar artery; VAS, Visual Analogue Scale; VBD, vertebrobasilar dolichoectasia; vs., versus.

^a^ Mann–Whitney *U* test.

^b^ Chi‐square test.

^c^ Adjusted Chi‐squared test.

^d^ Fisher^’^s exact test.

^e^ Independent samples *t*‐test.

No significant differences were observed between HCs and migraine patients in age, gender, BMI (Body Mass Index), or proportions of smokers and drinkers, except for sleep disturbance (HC vs. EM: *p* = 0.018; HC vs. CM: *p* < 0.001). When comparing EM and CM groups, no notable differences were observed in age, sex, BMI, smoking/drinking habits, age of onset, disease duration, or VAS scores. However, significant differences were observed in sleep disturbance (*p* < 0.001), migraine frequency (*p* < 0.001), MIDAS score (*p* < 0.001), HIT‐6 score (*p* = 0.012), PHQ‐9 score (*p* < 0.001), and GAD‐7 scores (*p* = 0.010).

### 3.2 MRI Features

**FIGURE 2:**
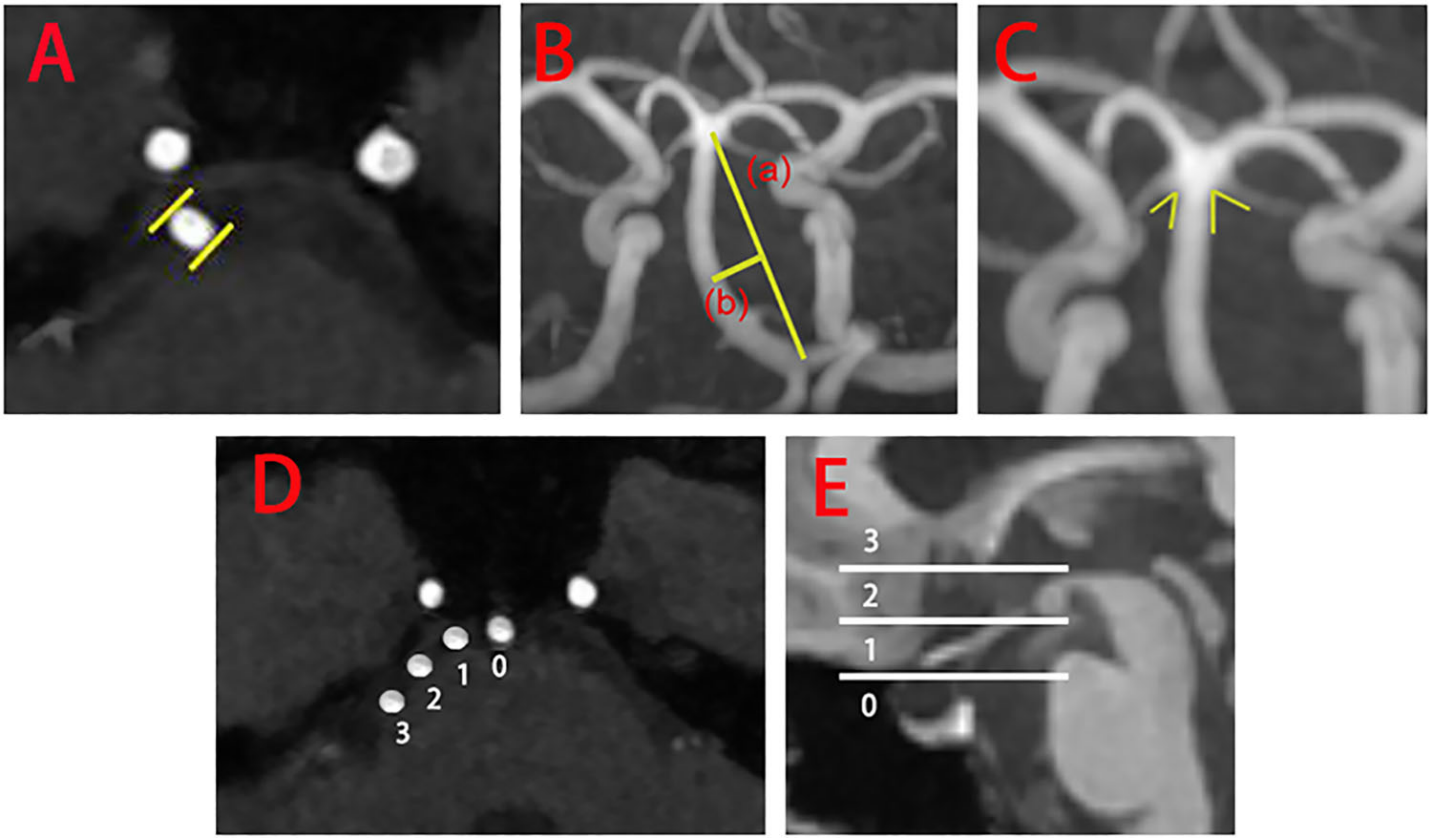
Demonstration of the assessment of the basilar artery. (A) Measurement of the maximum diameter of the basilar artery (the distance between two line segments). (B) Measurement of basilar artery length (segment a) and basilar artery lateral displacement (segment b). (C) Measurement of the superior cerebellar artery outlet angle (the sum of the two included angles). (D) Grades of basilar artery lateral displacement. (E) Grades of height of basilar artery bifurcation (the detailed criteria for grade classification are described in the methodological references).

The MRI example images for evaluated BA are displayed in Figure 2, and the distribution and grades of BALD and height of BA bifurcation are presented in Table 2.

**TABLE 2:** Distribution and grades of BALD and height of BA bifurcation.

	**HCs (*n* = 41)**	**EM (*n* = 41)**	**CM (*n* = 95)**
Displacement, *n* (%)			
Degree 0	28 (68.29)	18 (43.90)	38 (40.00)
Degree 1	12 (29.27)	22 (53.66)	51 (53.68)
Degree 2	1 (2.44)	1 (2.44)	3 (3.16)
Degree 3	0	0	3 (3.16)
Height of BA bifurcation, *n* (%)			
Degree 0	23 (56.10)	19 (46.34)	24 (25.26)
Degree 1	18 (43.90)	22 (53.66)	68 (71.58)
Degree 2	0	0	3 (3.16)
Degree 3	0	0	0

Abbreviations: BA, basilar artery; BALD, basilar artery lateral displacement; CM, chronic migraine; EM, episodic migraine; HCs, healthy controls.

We found no significant differences in BA diameter, BA length, BADE, and VBD between patients with EM or CM and the HC group, while significant differences were observed in BALD (HC vs. CM: *p* = 0.001) and SUCA outlet angle (HC vs. EM: *p* = 0.014; HC vs. CM: *p* = 0.001). No significant differences were observed in BA diameter, BA length, VBD, BALD, and SUCA outlet angle between EM and CM, except for BADE (*p* = 0.030).

### 3.3 Associations of Migraine and Features of BA

To establish a logistic regression model, we first conducted univariate analysis to screen predictor variables and only retained those with significant effects. Using the HC group as the reference category, we constructed a multinomial logistic regression model to assess the correlation between BA‐related indicators and EM or CM. The results were listed in Tables 3 and 4, respectively.

**TABLE 3:** Association between basilar artery and migraine by logistic regression analysis.

	**Crude OR (95% CI)**	***p* value**	**Adjusted OR (95% CI)**	***p* value**
Female	0.746 (0.348–1.599)	0.452	1.050 (0.440–2.509)	0.912
Age	1.023 (0.987–1.059)	0.214	0.990 (0.949–1.033)	0.655
Sleep disturbance	9.979 (3.688–27.004)	< 0.001	12.648 (4.351–36.771)	< 0.001
BALD	1.286 (1.069–1.549)	0.008	1.232 (1.011–1.501)	0.039
SUCA outlet angle	0.974 (0.958–0.991)	0.003	0.969 (0.948–0.989)	0.003

*Note: p* value of the Hosmer–Lemeshow test, 0.283.

Abbreviations: BALD, basilar artery lateral displacement; CI, confidence interval; OR, odds ratio; SUCA, superior cerebellar artery.

**TABLE 4:** Model of multinomial logistic regression, taking healthy control as the reference category.

**Comparison and covariate**	***β***	**SE**	**OR (95% CI)**	***p* value**
HC vs. EM				
Female	0.159	0.520	1.172 (0.423–3.246)	0.760
Age	−0.021	0.025	0.979 (0.933–1.027)	0.385
Sleep disturbance	−1.584	0.611	0.205 (0.062–0.679)	0.009
BALD	0.141	0.115	1.152 (0.919–1.443)	0.219
SUCA outlet angle	−0.031	0.012	0.970 (0.948–0.992)	0.009
HC vs. CM				
Female	−0.222	0.487	0.801 (0.308–2.081)	0.649
Age	< 0.001	0.024	1.000 (0.955–1.047)	0.997
Sleep disturbance	−3.058	0.574	0.047 (0.015–0.145)	< 0.001
BALD	0.260	0.109	1.297 (1.047–1.606)	0.017
SUCA outlet angle	−0.033	0.011	0.968 (0.946–0.990)	0.004

Abbreviations: *β*, beta; BALD, basilar artery lateral displacement; CI, confidence interval; CM, chronic migraine; EM, episodic migraine;

HC, healthy control; OR, odds ratio; SE, standard error; SUCA, superior cerebellar artery; vs., versus.

Our results suggested that migraine (both EM and CM) was significantly associated with sleep disturbance. After adjusting for age, sex, and sleep disturbance, migraine was significantly associated with both BALD (odds ratio [OR]: 1.232; 95% confidence interval [95% CI]: 1.011–1.501; *p* = 0.039, Table 3) and SUCA outlet angle (OR: 0.969; 95% CI: 0.948–0.989; *p* = 0.003, Table 3). In multinomial logistic regression, after adjusting for covariates, compared to the HC group, EM was significantly associated with SUCA outlet angle (OR: 0.970; 95% CI: 0.948–0.992; *p* = 0.009, Table 4), while CM was also significantly associated with both BALD (OR: 1.297; 95% CI: 1.047–1.606; *p* = 0.017, Table 4) and SUCA outlet angle (OR: 0.968; 95% CI: 0.946–0.990; *p* = 0.004, Table 4).

## 4 Discussion

The purpose of this study was to evaluate the relevant indicators of the BA in MO. Our main findings were as follows: (1) Patients with CM showed a significantly higher prevalence of severe BALD compared to the HC group, whereas no significant difference was observed in EM patients. (2) Both EM and CM patients had a significantly higher prevalence of reduced SUCA outlet angle than the HC group, with no significant difference between EM and CM. (3) Regarding tortuosity and elongation, CM patients had a significantly higher prevalence of BADE than EM patients. Multinomial logistic regression analysis revealed that both EM and CM were associated with SUCA outlet angle, while BALD was only associated with CM.

In the vast majority of healthy individuals, the BA runs straight along the midline or paramedian line (Smoker et al. 1986). Our findings may be explained through the role of matrix metalloproteinases (MMPs) in migraine pathophysiology. As key regulators in central nervous system disorders (Yong et al. 1998, 2001), MMPs degrade the internal elastic lamina, facilitating smooth muscle cell migration (Lehoux et al. 2006; Meng et al. 2007). This slow degenerative process could account for the increased BALD observed in CM. Abnormal vascular anatomy may influence migraine pathogenesis through dual mechanisms. From a hemodynamic standpoint, vertebrobasilar system curvature can alter distal flow dynamics at vascular confluences (Wake‐Buck et al. 2012). From a neurovascular viewpoint, posterior fossa vascular anomalies may mechanically irritate the trigeminal nerve, potentially activating trigeminovascular pathways (Hamlyn 1997). But apparently, the deviation of BA did not result in the nerve compression that would otherwise have caused trigeminal neuralgia. In stroke, BA displacement has been associated with hemodynamic changes that may precipitate cerebral ischemia (Kumral et al. 2005). Given the observed differences in cerebral blood flow within BA‐supplied regions between CM patients and HCs (Bai et al. 2022), we postulate BALD may contribute to migraine chronification through hemodynamic alterations.

We observed reduced SUCA outlet angles in both EM and CM patients. Multiple studies have identified decreased SUCA outlet angle as a marker for vertebrobasilar artery elongation (Laforêt et al. 2008; Montagnese et al. 2016). Intracranial arterial dolichoectasia may represent a common pathological pathway of arterial wall responses to diverse pathological mechanisms or injuries, with MMPs, particularly MMP‐3, potentially playing a key role in this process (Pico et al. 2015). Notably, MMP alteration has been documented in migraine patients. During the interictal period, elevated plasma MMP‐9 levels and cortical spreading depression (CSD) may synergistically enhance MMP upregulation and vascular permeability changes (Gursoy‐Ozdemir et al. 2004; Imamura et al. 2008). Concurrently, increased serum elastase could promote extracellular matrix degradation, potentially driving vertebrobasilar elongation (Dai et al. 2015; Tzourio et al. 2000). Interestingly, plasma MMP‐3 levels decrease during migraine attacks (Ashina et al. 2010), while recent Mendelian randomization evidence implicates MMP‐3 as a migraine risk factor and potential therapeutic target (Xiong et al. 2024). Among established risk factors for dolichoectasia development, old age and male sex may play significant roles (Gutierrez et al. 2011). This contrasts sharply with migraine epidemiology, where peak prevalence occurs in adolescent and middle‐aged women (Ferrari et al. 2022), a demographic pattern consistently reflected in our dataset. Thus, although we observed statistical differences in BADE between EM and CM, we cannot confirm an association between BA abnormalities and dolichoectasia in migraine patients, as this vascular phenotype is more strongly linked to cerebrovascular events. Given the distinct alterations of MMP‐3 and MMP‐9 observed in migraine, both of which are potentially involved in dolichoectasia pathogenesis, these MMPs likely serve different functional roles in migraine pathophysiology. The specific mechanisms underlying these changes deserve further study.

As a primary headache disorder, migraine has not shown significant macroscopic imaging markers in previous studies, and few have explored MRA as an imaging marker for migraine. Previous structural MRI studies have demonstrated no significant macroscopic or mesoscopic structural differences between MA and MO (Matharu et al. 2003), nor significant white matter microstructural changes between EM and CM (Neeb et al. 2015). Therefore, in our view, it may suggest that BALD may represent an independent risk factor for CM progression, potentially serving as an MRA biomarker for chronicity. Meanwhile, a reduced SUCA outlet angle may constitute both a risk factor and a potential imaging biomarker for MO. Our findings may provide some new insights into migraine as a primary neurovascular disorder.

### 4.1 Strengths and Limitations

The strength of this study lies in the discovery of BA‐related imaging indicators, previously reported in MA, within a population suffering from MO. Moreover, some of these indicators may serve as imaging biomarkers for distinguishing MO or as a risk factor for MO.

Our study has several limitations. First, as a single‐center study conducted exclusively at Tiantan Hospital (National Center for Neurology), the enrolled participants typically presented with more severe headache burdens. This selection bias prevents us from determining whether our conclusions would remain consistent if replicated in multicenter studies, particularly those involving primary care settings. Second, given the visual assessment‐based methodology, simply increasing sample size or conducting multicenter studies may be insufficient. Future investigations should incorporate multi‐omics data integration and machine learning approaches to better characterize MRA alterations in MO populations. Finally, the cross‐sectional design precludes causal inferences between alterations in BALD and SUCA and migraine pathogenesis. Longitudinal studies are warranted to validate these observed associations.

## 5 Conclusion

Currently, few macroscopic imaging biomarkers are available for migraine diagnosis. Our data suggest that the reduced SUCA outlet angle may represent a risk factor for MO and could potentially serve as an imaging biomarker. Additionally, BALD may constitute an independent risk factor for CM and could function as an MRA biomarker for migraine chronicity.

## Author Contributions

The study was conceptualized and designed by M.T.Z., X.L., H.F.T., and Y.G.W. M.T.Z. and Y.S.P. conducted the initial data analysis. M.T.Z., X.L., and H.F.T. oversaw data quality control measures. All authors participated in clinical and MRI data collection. M.T.Z. drafted the initial manuscript, which was critically reviewed and revised by all authors until consensus on the final version was reached.

## Conflicts of Interest

The authors declare no conflicts of interest.

## Peer Review

The peer review history for this article is available at https://publons.com/publon/10.1002/brb3.70955.

## Supporting information



Table S1. The detailed MRI parameters in this study.

## Data Availability

All data that support the findings of this study are available upon reasonable request to the corresponding author.
