# Supplementary material for: Changes of Basilar Artery in Patients With Migraine: A Case‐Control Study Based on 3T MRI
Source: Brain Behav. 2025 Oct 20;15(10):e70955. doi: 10.1002/brb3.70955 (PMC12537844; doi:10.1002/brb3.70955)
Supplement: Supplementary file 1 — Table S1. The detailed MRI parameters in this study. [file BRB3-15-e70955-s001.doc]

Table S1. The detailed MRI parameters in this study

| Sequence Name | Orientation | Acquisition parameters |
| --- | --- | --- |
| T1w-BRAVO | 3D sagittal | TR = 8.48 ms, TE = 3.24 ms, FA = 15°, slice thickness = 1.0 mm, acceleration matrix = 256 × 256, FOV = 260 mm |
| MRA | 3D | TR = 18.00 ms, TE = 3.40 ms, FA = 15°, slice thickness = 0.0 mm, acceleration matrix = 512 × 512, FOV = 220 mm |
| TOF-MRA | 3D axial | TR = 18.00 ms, TE = 3.40 ms, FA = 15°, slice thickness = 1.2 mm, acceleration matrix = 512 × 512, FOV = 220 mm |

*Note*: T1w-BRAVO, T1-weighted brain volume; MRA, magnetic resonance angiography; TOF-MRA, time-of-flight MRA; TR, repetition time; TE, echo time; FA, flip angle; FOV, feld of view.
